# Stability of an adaptive hybrid community

**DOI:** 10.1038/srep28181

**Published:** 2016-06-21

**Authors:** A. Mougi

**Affiliations:** 1 Department of Biological Science, Faculty of Life and Environmental Science, Shimane University, 1060 Nishikawatsu-cho, Matsue 690-8504, Japan

## Abstract

Contrary to stable natural ecosystems, the classical ecological theory predicts that complex ecological communities are fragile. The adaptive switching of interaction partners was proposed as a key factor to resolve the complexity–stability problem. However, this theory is based on the food webs that comprise predator–prey interactions alone; thus, the manner in which adaptive behavior affects the dynamics of hybrid communities with multiple interaction types remains unclear. Here, using a bipartite community network model with antagonistic and mutualistic interactions, I show that adaptive partner shifts by both antagonists and mutualists are crucial to the persistence of communities. The results show that adaptive behavior destabilizes the dynamics of communities with a single interaction type; however, the hybridity of multiple interaction types within a community greatly improves the stability. Moreover, adaptive behavior does not create a positive complexity–stability relationship in communities with a single interaction type but it does in the hybrid community. The diversity of interaction types is predicted to play a crucial role in community maintenance in an adaptive world.

Nature creates complex ecological communities comprising diverse species and interactions, contrary to a theory that predicts a negative relationship between ecosystem complexity and stability^1^. A key hypothesized factor to resolve this complexity–stability debate is “adaptive partner shift”; herein, predators maximize the energy gain per unit foraging effort by behavioral or evolutionary shift in prey selection^2^,^3^. Contrary to earlier studies, this theory incorporates a realistic feature where network topologies, such as interaction strengths and links, are not static and temporally change by adaptation, thereby constructing an observed network structure in natural food webs and a positive complexity–stability relationship^2^. However, real communities comprise a hybrid of different interaction types rather than merely a single interaction type^4^,^5^,^6^,^7^. In addition, in mutualistic interactions, the interaction partners change over time^8^, thereby constructing a realistic network topology in natural mutualistic webs^9^. Recently, adaptive partner shifts by both antagonists and mutualists were predicted to greatly affect community dynamics^10^; however, this study considered simple communities comprising only a few species; thus, the manner in which adaptive partner shifts affect more complex communities with multiple interaction types remains unclear.

Here I show that adaptive partner shifts in both antagonistic and mutualistic interactions completely change the stability of the dynamics of complex communities. I consider a bipartite network as a conceivable realistic network by merging both antagonistic and mutualistic interactions^4^. Community complexity can be quantified by the number of species involved in a community web (*N*) and the probability that a pair of species is connected by an interaction link (*P*). By controlling the adaptation speed (*G*) and the proportion of mutualism within a community (*p*_*m*_), I examine the effects of adaptation and diversity of interaction types on community stability with a given community complexity (*P*, *N*). The community stability is measured as proportional persistence, where the mean number of species that remain at the end of simulations is divided by the initial species richness (see the Methods). The analysis, contrary to the prediction of an earlier study^2^, shows that adaptive partner shift destabilizes the dynamics of communities with a single interaction type as community complexity increases, thereby constructing a negative complexity–stability relationship. However, in the hybrid community, adaptive partner shift dramatically increases the community stability, thereby constructing a positive complexity–stability relationship. I propose that the diversity of interaction types fulfills a crucial role in community stability in an adaptive world where interacting species behave adaptively.

## Results

**Figure 1:**
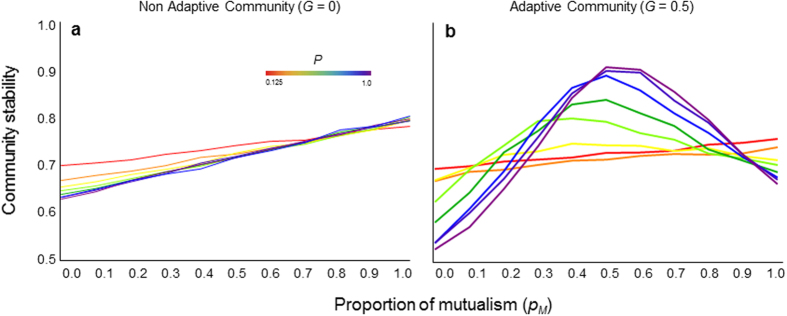
The relationships between the proportion of mutualism (*p*_*M*_) and stability. (**a**) Community without adaptation (*G* = 0). (**b**) Community with adaptation (*G* > 0). Colors indicate different levels of proportion of connected pairs *P*. I assume *N* = 20. See the Methods section for details of parameter values.

**Figure 2:**
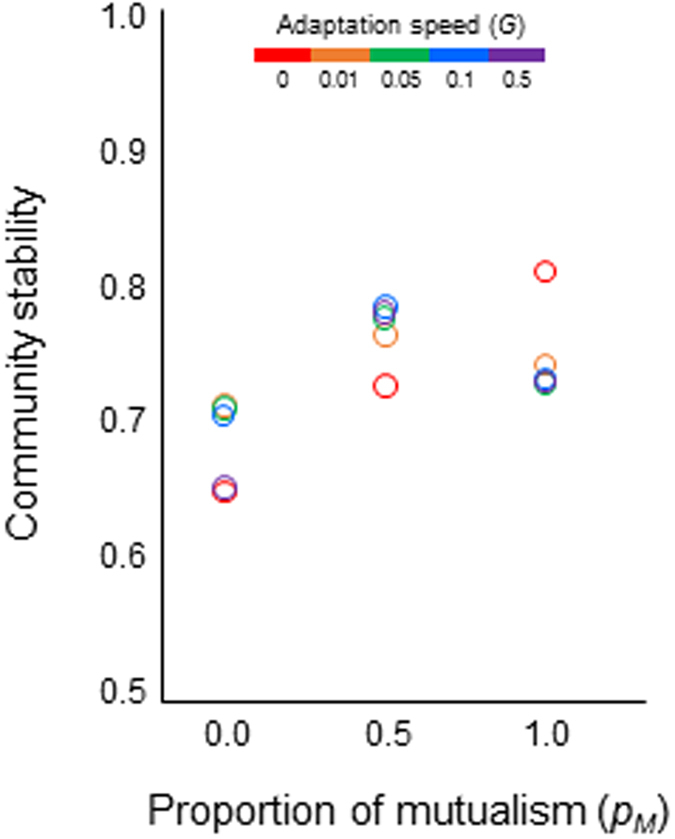
The effects of adaptation speed on the relationships between the proportion of mutualism (*p*_*M*_) and stability. Colors indicate the different levels of adaptation speed. I assume *N* = 16 and *P* = 0.5.

**Figure 3:**
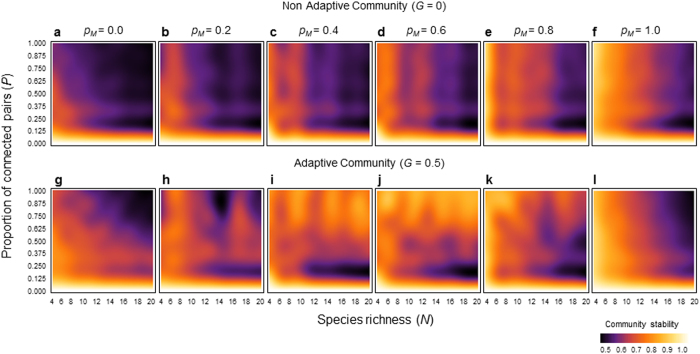
Complexity–stability relationships with varying proportions of mutualism (*p*_*M*_). (**a**–**f**) Community without adaptation (*G* = 0). (**g**–**i**) Community with adaptation (*G* > 0).

The considered communities comprise a single interaction type (*p*_*m*_ = 0 or 1). In both types of communities, adaptation tends to destabilize the population dynamics (Figs 1 and 2). The instability is higher when community complexity (*P* and *N*) is higher (Fig. 1, Fig. S1), indicating a negative relationship between community complexity and stability in the same way as in the case of no adaptation (Fig. 3a–f,g,l).

The mixing of both interaction types within a community dramatically changes the effects of adaptation on community dynamics in three ways. First, adaptation creates a stabilization effect due to the hybridity of multiple interaction types. Without adaptation, the hybridity of interaction types does not qualitatively change community stability (Fig. 1a, Fig. S1a). In contrast, with adaptation, the stability of a hybrid community is higher than that of a non-hybrid community and reaches a peak in an intermediate mixing of different interaction types (Fig. 1b, Fig. S1b). Second, faster adaptation enhances the stability of a hybrid community, contrary to that of a non-hybrid community (Figs 1 and 2). Third, adaptation can reverse an otherwise negative complexity–stability relationship of communities into a positive relationship (Fig. 3). The reversal is observed as long as different interaction types are mixed at intermediate levels. These results suggest that the diversity of interaction types plays a critical role in maintaining communities containing adaptive organisms.

## Discussion

Contrary to earlier theories^2^,^11^,^12^, adaptation causes instability in non-hybrid communities. In the random or cascade food webs assumed in the previous study^2^, adaptive exploiters are always likely to utilize more abundant species, resulting in the rescue of rare species, thereby causing a stabilization effect^2^. However, in a bipartite network, rare resource species are not likely to be rescued because (1) the consumers’ predation pressures on the resource species are inherently higher than random and cascade networks owing to the absence of top predators, (2) the interspecific competition between resource species promotes the extinctions of rare resource species utilized by consumers, and (3) partner shifts after the extinction of rare resource species increases the predation pressures on alternative resource species. This instability mechanism is supported by a recent theoretical study that used a model framework similar to that in the present study^13^. Mutualists additionally have a destabilization effect. Adaptive mutualists favor the more abundant resource species, thereby providing a mechanism of majority-advantage and thus potentially inhibiting the coexistence of resource species^10^. However, these instabilities caused by exploiters and mutualists can collectively result in a stabilization effect (Fig. S2). Adaptive exploiters rapidly begin to utilize abundant resource species that have benefited from mutualists, thereby avoiding competitive exclusion of less abundant resource species. Because of the reduction of abundant resources by exploiters, adaptive mutualists rapidly shift interacting partners to other resources, thereby promoting the rapid recovery of resource species. The stabilization mechanism, i.e., exploiters’ consuming more abundant resource species increased due to mutualists and rapid recovering effect on resource species due to mutualists, is known to work in a simple hybrid community system with a few species^10^, but will be more efficient in communities with more complexity (*N* and *P*) and adaptive organisms.

There are two basic ways to model hybrid communities with antagonistic and mutualistic interactions. The first way considers that antagonistic and mutualistic consumers are separate species sets^14^ [a species is either a mutualist (e.g., pollinator) or an antagonist (e.g., herbivore)]. The second way considers that a consumer can have both antagonistic and mutualistic interaction links^5^,^7^,^15^,^16^ (a consumer species can be both a pollinator and a herbivore, e.g., pollinating insects)^17^. Although the present study focused on the first scenario, we can speculate the consequences of the second scenario from the present study. If there are no costs to invest in mutualistic interactions, all consumer species would have mutualistic links because the increases in the resource species are always adaptive. In contrast, if the investments in mutualistic interactions are high, all consumers would have antagonistic links. These two extremes can destabilize the systems, as predicted by the present study. However, if the costs of mutualism are at a moderate level, each species would have both types of interactions, resulting in the stabilization of the systems, as predicted by the present study. In fact, a recent study supported this speculation^18^. In a system where pollinators play both roles of mutualistic and antagonistic interactions (pollinators are beneficial to the plants and consume the resources produced by the plants), adaptive foraging has a stabilizing role. These arguments suggest a general prediction that adaptation can stabilize hybrid communities with multiple interaction types.

An earlier theory showed that local stabilization effects due to interaction-type diversity need a negative relationship between the number of interactions and interaction strength^5^,^6^,^7^. The present study supports this theory from two perspectives. First, stabilization due to the hybridity of interaction types is successful within non-equilibrium dynamics. Second, an adaptive partner shift provides a mechanism for the specific assumption of a negative relationship between interaction strengths and the number of interaction links because each species allocate the interaction efforts to each interacting partner species and each interaction strength becomes weaker. In fact, in plant–pollinator–herbivore systems or plant–mycorrhizal symbiont–mycorrhizal parasite systems, partner shifts can occur adaptively^8^,^19^,^20^,^21^. The universal coexistence of mutualists and exploiters in natural communities may be maintained by adaptive partner shifts. In contrast, the switching of interaction types, such as shifts from mutualism to antagonism, can occur depending on environmental conditions^22^. The results of the present study warn about the possibility that a unidirectional adaptive shift in interaction types driven by human impacts^22^ results in the destabilization of community dynamics.

Stability has multiple components apart from proportional persistence^14^,^23^,^24^,^25^. In community dynamics research, we may need to capture various perspectives of stability. For example, persisting communities may be fluctuating over time. To consider this possibility, I also examined the mean of coefficient of variation (CV) of persisting populations as another stability measure. However, this system does not show population oscillations (Fig. S3), implying that persisting communities are stable in this system. In other words, adaptation increases persistence but weakly affects to temporal variability of population abundances. In addition, a previous study used local stability analysis, supporting the result that complexity destabilizes the bipartite mutualistic communities without adaptation^26^. However, another previous study using the same stability measure as the present study had a completely different prediction^27^. This discrepancy may be a result of the presence or absence of interspecific competition between resource species and nonrandom network structure (nestedness). In fact, nestedness and interspecific competition at each trophic level largely affect species coexistence^28^.

The present study links three components of biodiversity, namely genetic diversity, species diversity, and interaction-type diversity. Genetic diversity is an intra-species diversity that can increase the adaptation rate at a species level. Either component of diversity is not sufficient to support biodiversity by itself. Furthermore, a loss of a diversity component may result in a cascading loss of other diversity components. If genetic diversity is decreased because of any reason, the destabilization of the community can result, thereby causing species loss. The loss of species can cause not only a further decrease in community stability but also a decrease in interaction links and/or interaction types. The present study suggests that such a negative chain reaction continues to decrease the stability of ecosystems.

## Methods

Consider an ecological community comprising *N* species, where population dynamics is driven by interspecific interactions. The population dynamics of species *i* is described by the following ordinary differential equation:


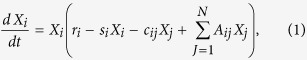


where *X*_*i*_ is the abundance of species *i*, *r*_*i*_ is the intrinsic rate of change in species *i*, *s*_*i*_ is density-dependent self-regulation, and *A*_*ij*_ is the interaction coefficient between species *i* and *j*. The bipartite network structure is used as an appropriate model with antagonistic and mutualistic interactions^4^. I assumed that interspecific competition between basal species (competition coefficients are annotated as *c*_*ij*_) can occur and that there is no direct interspecific competition between exploiters (*c*_*ij*_ = 0). Species numbers at each trophic level are the same. I defined the proportion of connected pairs *P* as the proportion of realized interaction links *L* in the possible maximum interaction links *L*_*max*_ of a given network model (*L* = *PL*_*max*_). In the bipartite network, *L*_*max*_ = (*N*/2)^2^ + (*N*/4)(*N*/2 − 1), where the first and second terms on the right-hand side represent the maximum links without interspecific competition between basal species and those of only interspecific competition between basal species, respectively. In the present study, only antagonistic and mutualistic links are controlled and competition links are fixed to (*N*/4)(*N*/2 − 1). The interaction coefficients *A*_*ij*_ and *A*_*ji*_ (*i* ≠ *j*) are defined as *A*_*ij*_ = *g*_*ij*_*α*_*ij*_ and *A*_*ji*_ = −*α*_*ij*_ in antagonistic interaction between the exploiter *i* and the resource *j*. These coefficients are defined as *A*_*ij*_ = *g*_*ij*_*α*_*ij*_ and *A*_*ji*_ = *g*_*ji*_*α*_*ji*_ in mutualistic interaction between the mutualist *i* and the resource *j*, where *g*_*ij*_ (<1) is the conversion efficiency when species *i* utilizes species *j*. If assuming a type II functional response, 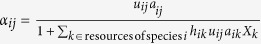, where *u*_*ij*_ is the potential interaction rate of species *i* on resource species *j*, defined as a per interaction rate when all interaction efforts are allocated to resource *j*; *h*_*ij*_ is the handling time; and *a*_*ij*_ is the interaction effort of species *i* allocated to resource *j* (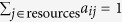 and *a*_*ij*_ = 0 when species *i* does not interact with species *j*). For simplicity, *g*_*ij*_ is set to a biologically feasible constant value and *g* = 0.15^29^,^30^. Simulations are iterated with randomly chosen parameter sets (*r*_*i*_ = 0.0–1.0, *u*_*ij*_ = 0.0–2.5, *c*_*ij*_ = 0.0–0.5, *h*_*ij*_ = 0.01, *s*_*i*_ = 1.0), initial abundances (*X*_*i*_ = 0.0–1.0), and linkage patterns. I confirmed that some relaxation of the symmetry of parameter values (*g*_*ij*_ = 0.0–0.3 or *h*_*ij*_ = 0.01–0.1) does not change the main results.

The dynamics of the interaction effort of an adaptive exploiter or mutualist *i* to resource species *j* (*a*_*ij*_) is given by a replicator equation:


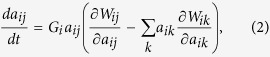


where *W*_*ij*_ is the per-capita growth rate of the exploiter or mutualist (*dX*_*i*_/*dt*) and *G*_*i*_ is the adaptation rate, which is higher when the adaptation is phenotypic plasticity or behavior and lower when it is evolutionary change. For simplicity, adaptation rates are set to a constant to all species (*G*).

In each simulation (iterated in 10^3^ runs), the number of species that remain at the end of simulations divided by the initial species is calculated after sufficiently long periods to reach asymptotic dynamics (*t* = 5 × 10^3^), and the average over the number of simulations is used as index of community stability^31^. The threshold of extinctions is defined as *X*_*i*_ < 10^−5^. An additional stability index is community persistence, which indicates the probability that all species persist for a given time in fluctuating environments^2^; however, this index is not used here because the persistence of all species is difficult in this system.

## Additional Information

**How to cite this article**: Mougi, A. Stability of an adaptive hybrid community. *Sci. Rep.* **6**, 28181; doi: 10.1038/srep28181 (2016).

## Supplementary Material

Supplementary Information
