# Author Correction: Stability of an adaptive hybrid community

**DOI:** 10.1038/s41598-018-30762-6

**Published:** 2018-08-10

**Authors:** Akihiko Mougi

**Affiliations:** Department of Biological Science, Faculty of Life and Environmental Science, Shimane University, 1060 Nishikawatsu-cho, Matsue, 690-8504 Japan

Correction to: *Scientific Reports* 10.1038/srep28181, published online 21 June 2016

The original version of this Article contained a typographical error in the spelling of the author Akihiko Mougi, which was incorrectly given as A. Mougi. This has now been corrected in the PDF and HTML versions of the Article, and in the accompanying Supplementary Information file.

