# Supplementary material for: Stability of an adaptive hybrid community
Source: Sci Rep. 2016 Jun 21;6:28181. doi: 10.1038/srep28181 (PMC4914837; doi:10.1038/srep28181)

**Supplementary Information**

# Stability of an adaptive hybrid community

A. Mougi

**Figure S1.** The relationships between the proportion of mutualism ($p_M$) and stability. (a) Community without adaptation ($G$ = 0). (b) Community with adaptation ($G$ > 0). Colors indicate different levels of species richness $N$. I assume $P$ = 0.625. See the Methods section for details of parameter values.

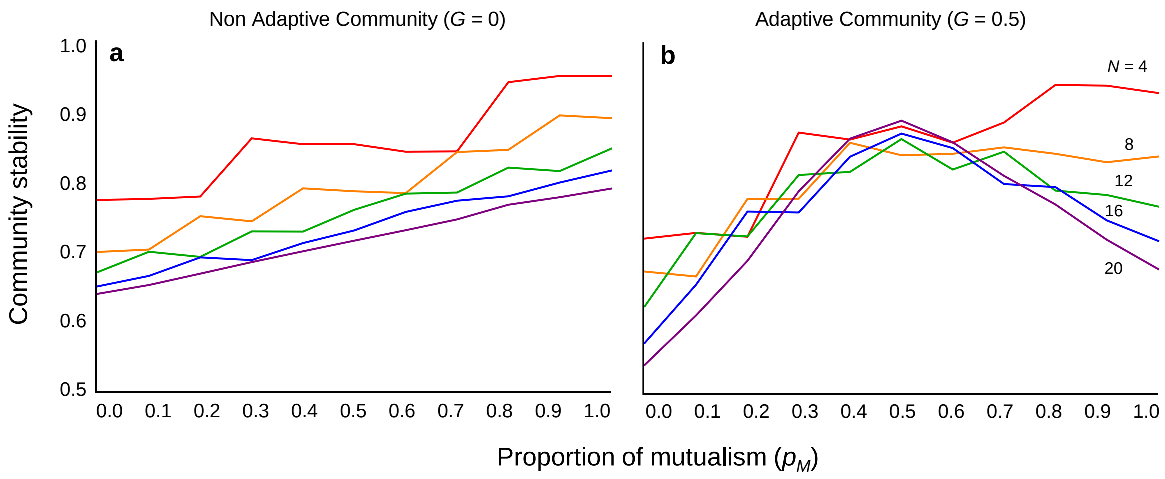

**Figure S2.** Typical network structures after sufficiently long simulation runs. (a) Non-hybrid communities. (b) Hybrid community. Red, blue, and green circles indicate antagonists, mutualists, and resource species, respectively. Dark green circles indicate the resource species that become extinct. Black lines indicate interaction links with large values (> $10^{-3}$).

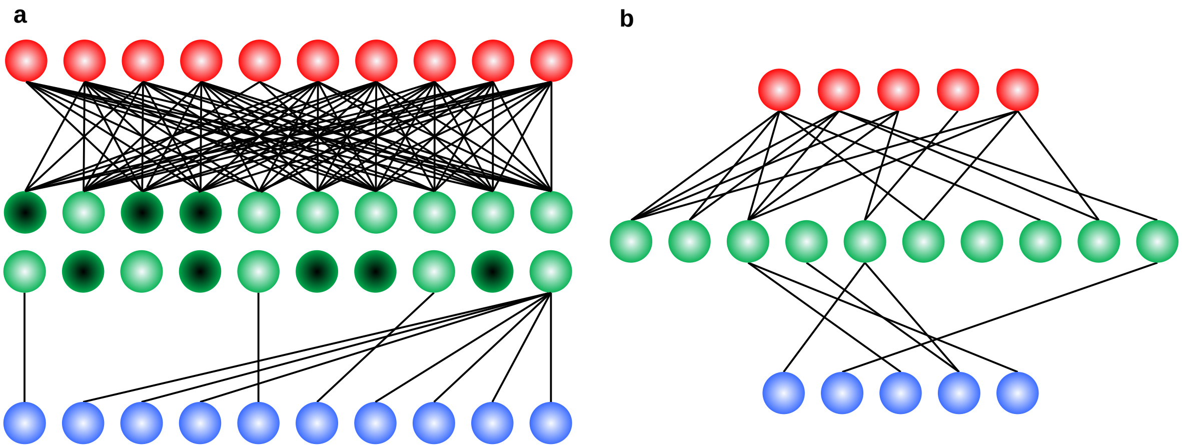

**Figure S3.** The relationships between the proportion of mutualism ($p_M$) and mean of coefficient of variation of persisting populations. (a) Community without adaptation ($G$ = 0). (b) Community with adaptation ($G$ > 0). Different colors indicate different values of $N$ ($N$ = 4, 8, 16) (in all cases, CV is very small). I assume $P$ = 0.5. See the Methods section for details of parameter values.

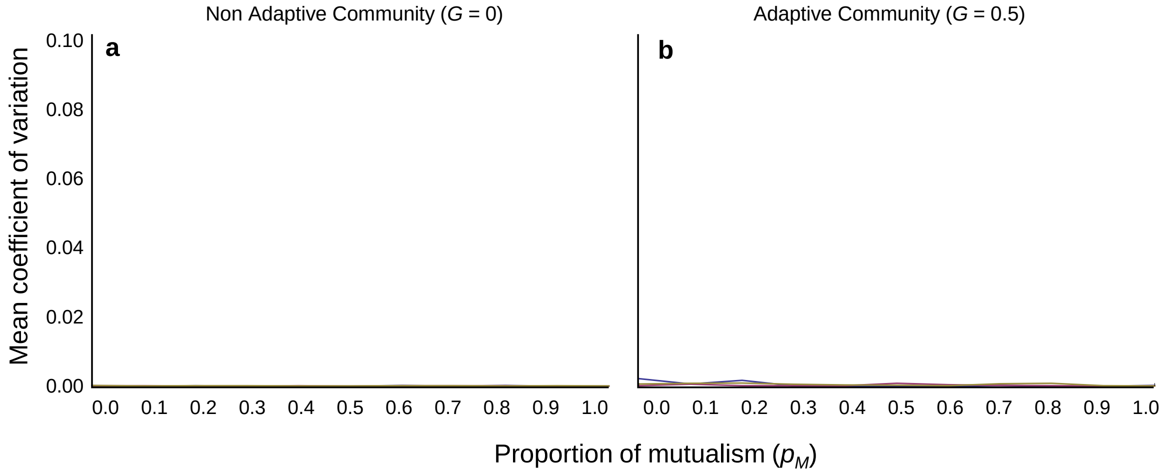

Supplement: Supplementary Information [file srep28181-s1.pdf]
